# Detection of Cryptic Fragile X Full Mutation Alleles by Southern Blot in a Female and Her Foetal DNA via Chorionic Villus Sampling, Complicated by Mosaicism for 45,X0/46,XX/47,XXX

**DOI:** 10.3390/genes12060798

**Published:** 2021-05-24

**Authors:** Alison Pandelache, David Francis, Ralph Oertel, Rebecca Dickson, Rani Sachdev, Ling Ling, Dinusha Gamage, David E. Godler

**Affiliations:** 1 Victorian Clinical Genetics Services, Murdoch Children’s Research Institute, Royal Children’s Hospital, Melbourne, VIC 3052, Australia; alison.arvaj@vcgs.org.au (A.P.); david.francis@vcgs.org.au (D.F.); ralph.oertel@vcgs.org.au (R.O.); 2 Maternal Fetal Medicine Department, Royal Hospital for Women, Randwick, NSW 2031, Australia; rebecca.dickson@health.nsw.gov.au; 3 Centre for Clinical Genetics, Sydney Children’s Hospital, Randwick, NSW 2031, Australia; rani.sachdev@health.nsw.gov.au; 4 Diagnosis and Development, Murdoch Children’s Research Institute, Royal Children’s Hospital, Melbourne, VIC 3052, Australia; ling.ling@mcri.edu.au (L.L.); dinusha.gamage@mcri.edu.au (D.G.); 5 Faculty of Medicine, Dentistry and Health Sciences, Department of Paediatrics, University of Melbourne, Parkville, VIC 3052, Australia

**Keywords:** fragile, X syndrome, FMR1, mosaicism, CGG, cultured chorionic villus, sample, southern blot, full mutation, 45,X0/46,XX/47,XXX

## Abstract

We describe a female with a 72 CGG *FMR1* premutation (PM) (CGG 55–199) and family history of fragile X syndrome (FXS), referred for prenatal testing. The proband had a high risk of having an affected pregnancy with a full mutation allele (FM) (CGG > 200), that causes FXS through hypermethylation of the *FMR1* promoter. The CGG sizing analysis in this study used AmplideX triplet repeat primed polymerase chain reaction (TP-PCR) and long-range methylation sensitive PCR (mPCR). These methods detected a 73 CGG PM allele in the proband’s blood, and a 164 CGG PM allele in her male cultured chorionic villus sample (CVS). In contrast, the Southern blot analysis showed mosaicism for: (i) a PM (71 CGG) and an FM (285–768 CGG) in the proband’s blood, and (ii) a PM (165 CGG) and an FM (408–625 CGG) in the male CVS. The *FMR1* methylation analysis, using an EpiTYPER system in the proband, showed levels in the range observed for mosaic Turner syndrome. This was confirmed by molecular and cytogenetic karyotyping, identifying 45,X0/46,XX/47,XXX lines. In conclusion, this case highlights the importance of Southern blot in pre- and postnatal testing for presence of an FM, which was not detected using AmplideX TP-PCR or mPCR in the proband and her CVS.

## 1. Introduction

Fragile X syndrome (FXS) is the most common single gene disorder associated with inherited disability and autism, affecting 1:4000 males and 1:5000–8000 females [1]. It is usually caused by the cytosine-guanine-guanine (CGG) expansion ≥200 repeats referred to as a full mutation (FM) in the 5′UTR end of the *FMR1* gene. This FM expansion usually leads to an abnormal methylation of the *FMR1* promoter that encompasses the CpG island located in the 5′ of the CGG repeat, as well as *FMR1* exon 1 and a portion of the *FMR1* intron 1, both located in the 3′ of the CGG expansion [2]. Methylation of this locus is also affected by X-inactivation in females [3] and has been associated with silencing of *FMR1* transcription and loss of fragile X mental retardation protein (FMRP), which is essential for normal neurodevelopment [4,5]. The levels of methylation at this locus have also been associated with intellectual functioning in both males [6] and females [7,8] affected with FXS. Moreover, abnormal methylation of the *FMR1* promoter is not restricted to FM alleles, with changes in DNA methylation associated with X-inactivation skewing in individuals affected with different types of sex chromosome aneuploidy [3], and in a proportion of males with idiopathic developmental delay with normal size (NS) (CGG < 45) or intermediate size (CGG 45–54) alleles [9].

The *FMR1* alleles with CGG sizes between 55 and 199 repeats, termed premutation (PM), are considerably more common than FM, found in approximately one in 300 females and approximately one in 800 males, in the general population [10]. These alleles usually have a completely unmethylated *FMR1* promoter. The PM alleles in females have also been associated with an increased risk of having a child affected with FXS, with this risk increases proportionally with an increase in the CGG size of the maternal PM allele [11]. Adenine-guanine-guanine (AGG) interruptions (found at the 5′ end of the CGG expansion) have also been suggested to modify the chance of expansion [12]. This information is used to counsel couples requesting prenatal diagnosis of FXS [13,14].

Typically, prenatal testing involves sex determination by karyotype and fluorescence in situ hybridization (FISH), and CGG sizing using routine polymerase chain reaction (PCR) and Southern blot analysis [15]. Triplet repeat primed PCR (TP-PCR) and long-range PCR, using commercial kits for *FMR1* CGG screening and linkage techniques, have also been used in these settings [16]. In contrast, *FMR1* methylation analysis on proband and/or foetal DNA is not typically performed as part of prenatal testing for FXS, despite being suggested to be informative in mosaic cases in these settings [17,18].

## 2. Clinical Report

### 2.1. Clinical History and Consent

A twenty-six-year-old female with a family history of FXS was referred for prenatal testing due to a family history of FXS. Her initial results found a normal size allele (<44 CGGs) of 30 CGG repeats and a 73 CGG PM allele detected by AmplideX triplet repeat primed PCR (TP-PCR) sizing after cascade carrier testing. The proband’s maternal cousin’s three children were previously found to be carrying PM and FM alleles. The proband’s mother was identified to have a 37 CGG and 59 CGG PM as part of the cascade testing by AmplideX TP-PCR. The proband did not have any history of developmental delay and was neurotypical. The proband’s height was 152 cm on the 3rd centile and she reported that she had a “hole in the heart” at birth and a unilateral left solitary kidney after removal of her right polycystic kidney in childhood. She also reported being treated for hypothyroidism and oligomenorrhea and had difficulty conceiving. Initial prenatal testing involved TP-PCR on fetal DNA via chorionic villus sampling (CVS) at 12 weeks plus 2 days gestation, and identified a single 156 CGG PM with an atypical stutter pattern up to 170 CGG. This raised concern that the allele represented a biased PCR result with a possibility of mosaicism for FM alleles. The case was then reflexed for Southern blot analysis [19].

Verbal consent was obtained from the proband when her samples were submitted for the follow-up clinical testing at the Victorian Clinical Genetics Services for her anonymized clinical information and fragile X testing results to be used in this study. For these reasons, additional ethical approval was not required.

### 2.2. Sample Processing

Maternal DNA was extracted from ethylenediaminetetraacetic acid (EDTA) preserved blood and DNA was extracted from a CVS from a local laboratory. Further proband testing of established phytohaemagglutinin (PHA) stimulated peripheral blood lymphocyte cultures was performed, with a harvest of metaphase chromosomes using a standard laboratory procedure, as previously described [20]. The Giemsa-banded analysis of 60 cells was examined to produce a conventional karyotype analysis. The molecular karyotype analysis was also performed, as previously described [18].

### 2.3. FMR1 CGG Sizing

Routine *FMR1* testing involved first-line PCR-based assessment of CGG repeat size using a validated PCR assay, with the upper limit of detection of 170 CGGs in males and 157 CGGs in females [15]. All DNA samples that showed a CGG size in the PM range, or failed to show a PCR product, were referred for second-line confirmatory testing by Southern blotting, using a 520-bp probe segment (Pfxa3) of the 1-kb Pst 1 fragment and ps8 control, as previously described [19]. Two different triplet primed long-range PCR commercial kits were also used for CGG sizing, i.e., AmplideX TP-PCR and AmplideX mPCR (Asuragen, Austin, TX, USA), performed as previously by [21,22], and on the same DNA samples as those analyzed by Southern blot. The AmplideX TP-PCR has a precision of ±2 repeat error, either classified as low (NS-54 CGG) or as increased risk (55–200 CGG or >200 CGG) determined using Gene Mapper 5.0 (Thermo Fisher Scientific, Waltham, MA, USA), as previously by [21].

**Figure 1:**
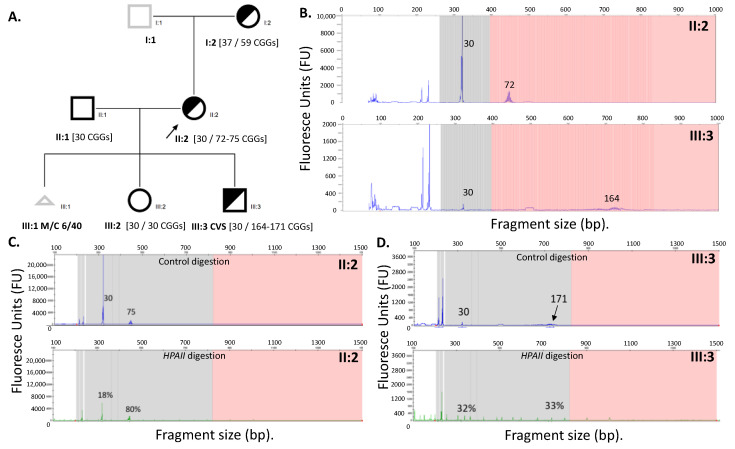
(**A**) Pedigree of the studied family with CGG sizing determined using an AmplideX TP-PCR. Males are represented by squares, females by circles and triangle sex unknown. A black outline indicates examined and half shaded indicates carrier. A black arrow indicates proband. III:1 Miscarriage at 6 of 40 weeks; (**B**) AmplideX TP-PCR CGG sizing, with no FM alleles detected in either III:3 male CVS or II:2 proband; (**C**) II:2 and (**D**) III:3 AmplideX mPCR, with the upper panel in blue representing CGG sizing based on control digestions and the lower panel in green representing relative methylation based on *HpaII* methylation sensitive restriction enzyme-based digestion followed by long-range PCR. No FM alleles are detected in either III:3 male CVS or II:2 female. Skewed methylation for the 75 CGG PM allele is suggested by 80% methylation. Note: numbers on each panel next to specific peaks indicate CGG sizes.

Confirmatory testing utilized a combination of standard CGG sizing PCR [15], AmplideX based analysis, and Southern blot, to exclude the presence of FM alleles in the CVS sample. CGG sizing using AmplideX TP-PCR for the proband identified a 30 and a 72 CGG PM allele (II:2), and a 30 CGG and a 164 CGG PM allele in the male CVS (III:3) (Figure 1). The standard CGG sizing PCR [15] detected a 30 CGG and a 74 CGG allele in the proband (II:2) whilst it had failed amplification for the CVS sample (III:3).

The 30 CGG allele in the male CVS is likely a result of maternal cell contamination, as it is the same size as the normal size allele in the mother (Figure 1). Interestingly, its methylation is different in CVS (32%) as compared with the mother’s normal size allele (18%) (Figure 1C,D). One potential explanation could be that, by chance, the maternal cell contamination involved a greater proportion of cells that had the normal size allele on the inactive X chromosome as compared with maternal blood. Another explanation may be related to technical limits of the mPCR assay used for quantitative methylation analysis in a small proportion of cells associated with maternal contamination.

**Figure 2:**
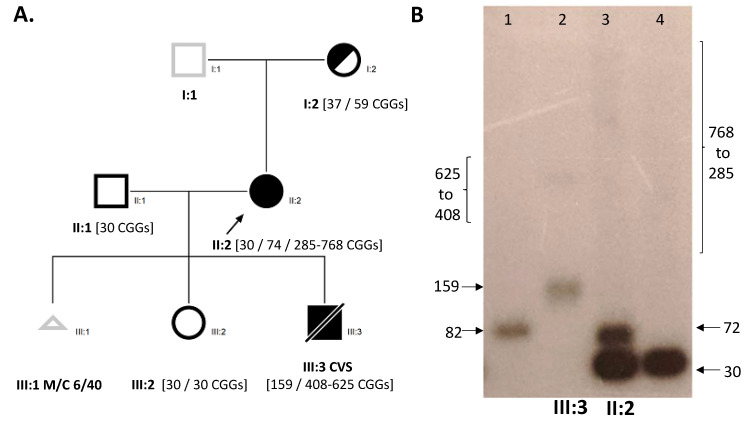
(**A**) Pedigree of the studied family with CGG sizing determined using Southern blot and standard CGG sizing PCR. Males are represented by squares, females by circles and triangle sex unknown. The black outline indicates examined, half shaded indicates carrier, and full shaded indicates affected with FXS. The black arrows indicate proband and a diagonal line indicates being deceased. III:1 Miscarriage at 6 of 40 weeks (**B**) Southern blot Lane 1 (male PM control), Lane 2 (III:3 male PM/FM CVS), Lane 3 (II:2 PM/FM female), Lane 4 (male normal CGG size control).

Importantly, no FM alleles were detected by AmplideX TP-PCR in both the proband and the CVS. In contrast, FM allele smears were detected in both the proband and male CVS using Southern blot (Figure 2B). On the basis of the Southern blot results, the proband chose to terminate the pregnancy. Unfortunately, tissues from the terminated foetus were not available to confirm these findings.

### 2.4. FMR1 Methylation Analysis

The *FMR1* methylation testing was performed for both CVS (III:3) and proband (II:2) using the EpiTYPER system targeting 12 CpG sites within the fragile X related epigenetic element 2 (FREE2) (located at the *FMR1* exon 1/intron 1 boundary) [23], and the AmplideX mPCR commercial kit targeting two *HpaII* restriction sites on either side of the CGG repeat (one within the CpG island and the other within FMR1 exon 1) [21]. For the CVS at 12 weeks of gestations, these analyses were performed after DNA methylation had been establishment (reported to occur at 11 weeks of gestation for the *FMR1* promoter in a human foetus) [24]. AmplideX mPCR showed that, in the proband *HpaII* sites, both the 30 and 171 CGG alleles were approximately 30% methylated. The proband’s X-inactivation skewing was observed with the 74 CGG allele being 80% methylated, whilst the 30 CGG allele was only 18% methylated. The AmplideX mPCR assay, however, did not detect an FM allele in either the proband (11:2) or CVS DNA (III:3) (Figure 1C).

The EpiTYPER methylation results in the CVS sample across 12 FREE2 CpGsites approached 0%; while for the proband FREE2 methylation approached 10%. This was an unexpected finding for the female proband, as this was significantly below the methylation levels typically observed for the same CpG sites in females with NS, PM, and FM alleles (Table 1). These methylation levels were, however, within the range described in females with mosaic Turner syndrome (24). 

### 2.5. Follow-Up Conventional Cytogenetic and Molecular Karyotype Analysis for II:2

**Figure 3:**
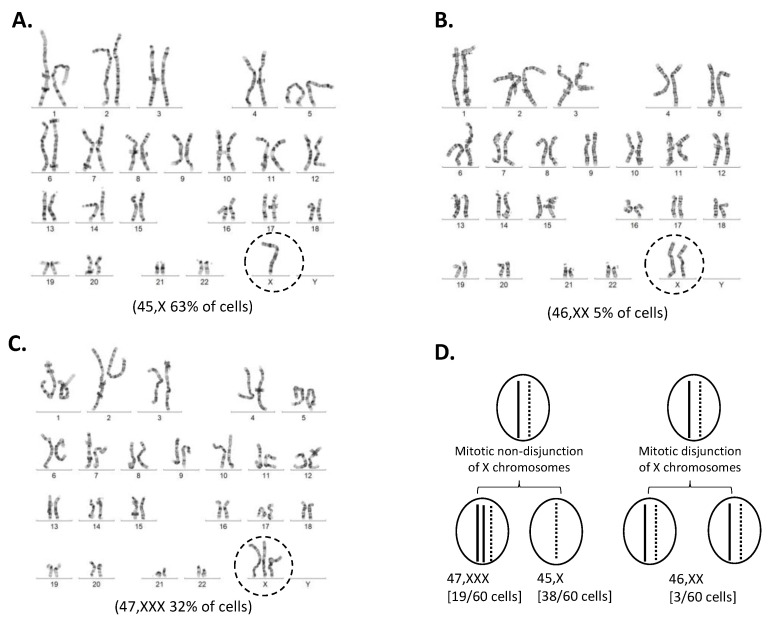
G banded chromosome analysis of II:2 proband blood and conventional karyotypes identify mosaic cell lines. (**A**) 45,X0 was found in 38/60 karyotypes analysed; (**B**) 47,XXX was found in 19/60 karyotypes analysed; (**C**) 46,XX was found in 3/60 karyotypes analysed. (**D**) Diagram of post zygotic nondisjunction and generation of the mosaic cell line 45,X[38]/47,XXX[19]1/46,XX[3]3.

**Figure 4:**
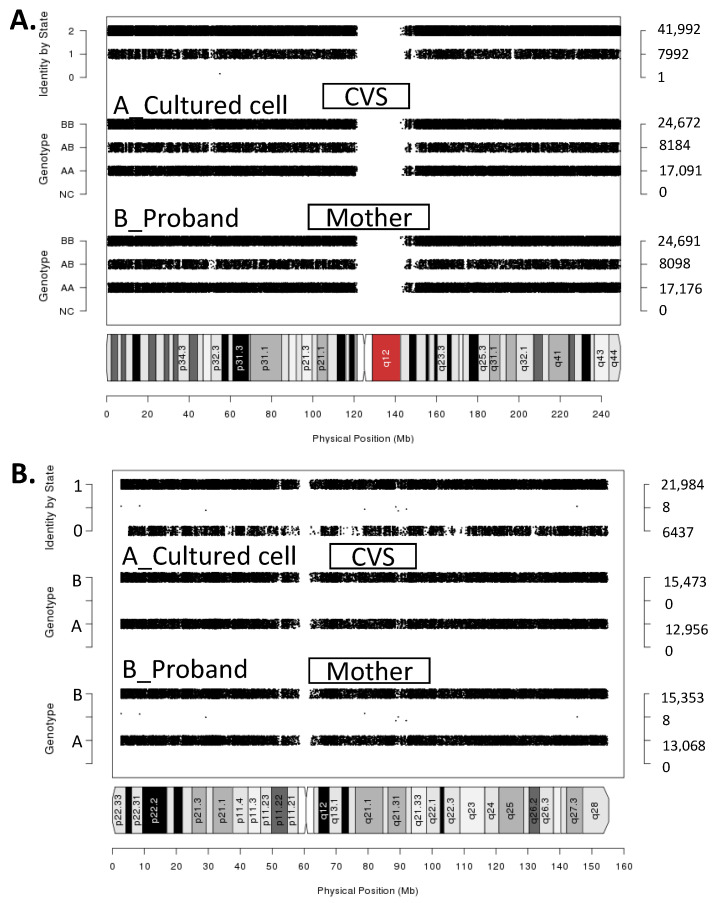
Linkage analysis using chromosomal microarray. (**A**) Single nucleotide polymorphisms (SNP) duo image for chromosome 1 showing identity by state (sharing one allele) for the whole chromosome. Whole genome comparison confirmed child-parent relationship. Note: Chromosome 1 SNPduo Output A_Culture CVS–B_Proband Mother Average Identity by State (IBS):1.84; (**B**) SNP duo for chromosome X between the foetus and the X0 cell line showing IBS0, that is sharing no alleles for most of the chromosome including the *FMR1* gene. Note: Chromosome X SNPduo Output A_Cultured CVS–B_Proband Mother Average IBS:1.1.547.

A conventional and molecular karyotype was requested by the referring doctor (Table 1, Figure 3 and Figure 4). A conventional cytogenetic karyotype analysis was performed using Giemsa/Trypsin/Leishman stain banding (G-banding) to produce visible staining of condensed metaphase chromosomes from venous blood. G-banding identified three different karyotypes in the proband including mosaicism for X0/XX/XXX cell lines, a female karyotype with sex chromosome mosaicism. Among the 60 cells examined, 38 cells (approximately 63%) showed a 45,X0 cell line, 19 cells (approximately 32%) showed a 47,XXX cell line, and the remaining three cells (approximately 5%) showed a normal female (46,XX) karyotype (Figure 3). The proband’s short stature, history of congenital heart disease, renal anomaly, hypothyroidism, and oligomenorrhea are clinically consistent with a 45,X0 phenotype. 

The molecular karyotype analysis of the whole genome was performed using chromosomal microarray (Figure 4). Single nucleotide polymorphisms (SPN) duogenotype comparisons were performed for the whole genome between the proband mother and foetus and showed sharing of one allele (identity by state 1), in over 97,600 informative probes used. This confirmed a parent-child relationship. Comparisons from chromosome 1 are shown in Figure 4 as a representation. The SNP duo genotype comparison between the mother’s 45,X cell line and the foetus’s abnormal X chromosomes were manually modified and the process of deduction confirmed that the abnormal X was found in the maternal 45,X0 cell line showing identity by state equaling zero. The father’s DNA sample was not available, and thus was not used in these analyses. 

Considering that the previously reported methylation of FREE2 for XXX females was ~43%, for X0 was 0%, and for XX was ~27% (Table 1), by combining these figures with proportions of each cell line observed in the blood of the female, from karyotyping, FREE2 methylation would equate to approximately 15% ((XXX: 43% methylation times 0.32) + (XX: 27% M times 0.05) + (X0: 0% methylation times 0.63)). This is consistent with the molecular karyotype analysis (Figure 4) and FREE2 methylation results observed for the proband (Table 1), providing a likely explanation for why FREE2 was hypomethylated in this female.

## 3. Conclusions

This study describes a femaleproband referred for prenatal testing due to a family history of FXS with 45,X0/46,XX/47,XXX cell lines, and mosaicism for PM and FM alleles. Although previous studies have reported 45,X0/46,XX mosaicism in FM females [26] and have suggested that it may be much more common than expected in females with a FM [27], until now, these cases have not been described in prenatal settings. Moreover, it has been suggested that the presence of an FM on an X chromosome may predispose it to loss during mitosis, and that this may occur due to structural changes of the metaphase chromosome with an FM [26,27,28].

Mosaicism for 45,X0/46,XX /47,XXX in the proband described in this report has implications for future genetic counselling regarding reproductive options and disease risks observed in mosaic Turner syndrome. It is important to note that it was initially flagged in this study through skewed X-inactivation patterns using FREE2 methylation testing, not typically performed in prenatal settings. This mosaicism was subsequently confirmed by microarray and karyotype analyses performed to identify the origins of this skewed X-inactivation. Conversely, mosaicism for PM and FM alleles found in the proband’s blood, and her male CVS have implications for both counselling related to FXS, as well as for future diagnostic testing. Specifically, for the male prenatal case, CVS and the proband mother FM alleles were missed by two independent commercial assays, AmplideX PT-PCR and AmplideX mPCR that are often used for prenatal and diagnostic testing for FXS [16]. This FM allele was detected in both samples using Southern blot analysis. Such a discordance may be explained by either or both primer sites for AmplideX TP-PCR and AmplideX mPCR assays being lost in this mosaic female and her CVS. Although the exact mechanism for this is unknown, this explanation is consistent with similar observations previously reported in a mosaic FXS male in post-natal settings, where one of the primer sites was lost due to a microdeletion proximal to the CGG expansion [29]. Altogether, these cases highlight the importance of Southern blot testing in prenatal and postnatal diagnostic testing for FXS.

## Figures and Tables

**Table 1:** Comparisons of the CpG10-12 FREE2 methylation for the male CVS and proband blood with the sex chromosome aneuploidy, typically developing female and male reference cohorts from earlier studies [3,17,25].

Group	Tissue	N	CGG Size	CMA ^d^	Meth. %	MAX %	MIN %
46,XY controls ^a^	Blood	14	<40		2 (±4)	4	0
46,XX ^b^ control	Blood	35	<40		27 (±10)	38	16
47,XXX ^c^	Blood	8	N/A	47,XXX	43 (±8)	47	38
45,Xo ^c^	Blood	11	<40	45,X	1 (±3)	4	1
(III:3)	CVS	1	159, 408–625	46,XY	3		
(II:2)	Blood	1	30, 72, 285–768	45,X/46,XX/ 47,XXX	8		

Note: Methylation % for reference samples is expressed as mean (±2 standard deviations). ^a^ Convenience sample of consenting typically developing males. ^b^ De-identified sample of females recruited in a population FXS carrier screening study. ^c^ De-identified sample taken as part of fragile X cascade testing and routine molecular microarray testing/karyotyping as part of previous studies. ^d^ Chromosomal microarray-based molecular karyotyping and standard karyotyping for III:3.

## Data Availability

The data that support the findings of this study are available on request from the corresponding author. The data are not publicly available due to privacy or ethical restrictions.

## Abbreviations

FXS	Fragile X Syndrome
*FMR1*	Fragile X mental retardation 1
FMRP	Fragile X mental retardation protein
CGG	Cytosine-guanine-guanine
AGG	Adenine-guanine-guanine
PCR	Polymerase chain reaction
CVS	Chorionic villus sampling
FISH	Fluorescence in situ hybridization
PM	Premutation
FM	Full mutation
SNP	Single nucleotide polymorphism
TP-PCR	Triplet repeat primed PCR
